# Longitudinal Trend of Plasma Concentrations of Extracellular Vesicles in Patients Hospitalized for COVID-19

**DOI:** 10.3389/fcell.2021.770463

**Published:** 2022-01-17

**Authors:** Elena Campello, Claudia Maria Radu, Chiara Simion, Luca Spiezia, Cristiana Bulato, Sabrina Gavasso, Daniela Tormene, Nicola Perin, Giacomo Turatti, Paolo Simioni

**Affiliations:** General Medicine and Thrombotic and Hemorrhagic Diseases Unit, Department of Medicine, Padova University Hospital, Padova, Italy

**Keywords:** microvesicles, hypercoaguability, inflammation, SARS–CoV–2, venous thomboembolism

## Abstract

Plasma concentrations of extracellular vesicles (EVs) originating from cells involved in COVID-19-associated coagulopathy (CAC), their longitudinal trend and association with clinical outcomes were evaluated. Blood samples of consecutive COVID-19 patients admitted to a medical Unit were longitudinally collected within 48 h of admission, at discharge and 30 days post-discharge. EVs were analyzed using high sensitivity flow cytometry and phospholipid-dependent clotting time (PPL). The following EVs were measured: endothelium-, platelet-, leukocyte-derived, bearing tissue factor (TF)+, angiotensin-converting enzyme (ACE2)+, platelet-derived growth factor receptor-β (PDGF-β)+ and SARS-CoV-2-nucleoprotein (NP)+. 91 patients were recruited for baseline EV analysis (mean age 67 ± 14 years, 50.5% male) and 48 underwent the longitudinal evaluation. From baseline to 30-days post-discharge, we observed significantly decreased plasma concentrations of endothelium-derived EVs (E-Selectin+), endothelium-derived bearing TF (E-Selectin+ TF+), endothelium-derived bearing ACE2 (E-Selectin+ACE2+) and leukocyte-EVs bearing TF (CD45+TF+), *p* < 0.001, *p* = 0.03, *p* = 0.001, *p* = 0.001, respectively. Conversely, platelet-derived (P-Selectin+) and leukocyte-derived EVs (CD45+) increased from baseline to 30-days post-discharge (*p* = 0.038 and 0.032, respectively). EVs TF+, ACE2+, PDGF-β+, and SARS-CoV-2-NP+ did not significantly change during the monitoring. PPL increased from baseline to 30-days post-discharge (+ 6.3 s, *p* = 0.006). P-Selectin + EVs >1,054/µL were associated with thrombosis (*p* = 0.024), E-Selectin + EVs ≤531/µL with worsening/death (*p* 0.026) and 30-days P-Selectin+ and CD45 + EVs with persistent symptoms (*p* < 0.0001). We confirmed increased EVs originating from cells involved in CAC at admission and discharge. EVs derived from activated pericytes and expressing SARS-CoV-2-NP were also detected. 30-days post-discharge, endothelium-EVs decreased, while platelet- and leukocyte-EVs further increased, indicating that cellular activation persists long after the acute phase.

## Introduction

A hypercoagulable state is commonly considered a major component of the pathophysiology of the infectious disease (COVID-19) caused by the novel severe acute respiratory syndrome coronavirus 2 (SARS-CoV-2) (Iba et al., 2020a; Connors and Levy, 2020; Görlinger et al., 2020). COVID-19 is characterized by a dysregulation of multiple biological pathways, spearheaded by an abnormal immune response and a persistent pro-inflammatory state, which ultimately converge to trigger the development of a serious hemostasis disturbance in the form of localized and systemic coagulopathies and thrombotic events (Iba et al., 2020b; Connors and Levy, 2020; Lippi et al., 2021). Microvesicles (MVs) are extracellular vesicles (EVs) of a defined size range (100–1,000 nm in diameter) that originate from cytoplasmic membrane budding of activated cells and can be identified by cell-specific surface markers (van der Pol et al., 2016; Saheera et al., 2021). MVs are highly procoagulant due to the presence of negatively charged phospholipids (e.g., phosphatidylserine) on their membranes, thus facilitating the assembly of protease complexes of the clotting cascade. In some pathological conditions MVs may also express tissue factor (TF), the primary cellular initiator of blood coagulation that can directly trigger the extrinsic coagulation pathway (Owens and Mackman, 2011; Campello et al., 2016a; Mooberry and Key, 2016). The surface antigen makeup of each MV subtype depends on its cellular origin and the physiological (or pathophysiological) processes responsible for its release. Therefore, measuring the plasma concentrations of one specific MV could potentially provide diagnostic and prognostic information about silent conditions (Owens and Mackman, 2011; Mooberry and Key, 2016; van der Pol et al., 2016; Campello et al., 2016b). COVID-19 patients show many of the pathophysiological processes that are associated with the cellular release of EVs, including endothelial injury, platelet hyper-reactivity, TF-mediated procoagulant activity, and increased thrombin generation (Iba et al., 2020b; Spiezia et al., 2020; Campello et al., 2021a; Lippi et al., 2021). Currently, there are several publications on the possible role of EV in the pathogenesis of SARS-CoV-2 infection. Guervilly et al. found that EV-associated TF activity increased dramatically in patients with severe COVID-19 and correlated with an increased risk of thrombotic complications (Guervilly et al., 2021). Furthermore, Balbi et al. specifically characterized the surface antigen profile of EVs in COVID-19 patients using a combination of seven surface molecules (CD49e, CD209, CD86, CD133/1, CD69, CD142, and CD20) (Balbi et al., 2021). Cappellano et al. also showed that platelet-derived EVs were higher in Sars-Cov-2+ patients compared to Sars-Cov-2 patients or healthy controls (Cappellano et al., 2021). Very recently, it was also showed that COVID-19 patients exhibited significantly higher numbers of EVs derived from platelets, endothelial cells, leukocytes, or neutrophils than controls (Traby et al., 2021). Finally, Kudryavtsev et al. showed that EVs concentrations are severity-related, as patients with severe infections had lower levels of some EVs vs. healthy controls (i.e., CD4^+^, CD19^+^, and CD146^+^) (Kudryavtsev et al., 2021). On the other hand, there is currently no data on the trend of EV levels after COVID-19 remission.

Therefore, the goals of this study were (a) to confirm plasma concentrations of different EV subtypes originating from activated endothelial cells, platelets, leukocytes, pericytes, TF-bearing, and nucleoprotein (NP) SARS-CoV-2-bearing, as well as EV-associated procoagulant activity in patients hospitalized for COVID-19, and (b) evaluate the longitudinal trend of EV subtypes at discharge and 30 days post-discharge. Finally, (c) we investigated the role of EVs as biomarker to identify patients at higher risk of VTE and worsening/death.

## Material and Methods

### Patients

We enrolled all consecutive patients aged ≥18 years with mild to moderate COVID-19 admitted to the General Medicine COVID Unit at Padova University Hospital between November 1, 2020 and February 1, 2021. COVID-19 diagnosis was confirmed by real-time reverse transcriptase polymerase chain reaction (rRT-PCR) assay using nasopharyngeal swab specimens. Exclusion criteria were: admission from a ward other than the Emergency Department (ED), admission for venous thromboembolism, surgery in the previous month, known pre-existing congenital bleeding disorders, Child’s C liver disease, end-stage kidney disease, and ongoing anticoagulant therapy. Patients characteristics such as demographics, comorbidities, ongoing treatments, and main outcomes, were recorded. Routine blood chemistry and hematologic parameters, including blood count, C-reactive protein (CRP), procalcitonin (PCT), ferritin, and interleukin-6 (IL-6) were recorded. Primary hemostasis exploration included prothrombin time (PT), activated partial thromboplastin time (aPTT), fibrinogen, D-dimer, factor VIII, von Willebrand factor anti-gen and antithrombin.

All patients underwent lower limb veins ultrasonography during the hospital stay, while Doppler ultrasound of upper limb or pulmonary computed tomography (CT) scan were performed in the presence of clinical suspicion for venous thromboembolism (VTE) (i.e., symptoms or sudden increased D-dimer levels).

The protocol was approved by the local Institutional Ethical Committee (Ref: 5001/AO/21; AOP2155). The study was conducted in compliance with the principles of the Declaration of Helsinki.

### Blood Sampling for EV Analysis

Blood samples were longitudinally collected within 48 h of patient admission, at discharge and 30 days post-discharge. Blood was drawn by venipuncture directly into BD Vacutainer^®^ Citrate Tubes containing 0.109 M (3.2%) sodium citrate (9:1 blood to anticoagulant ratio). Platelet poor plasma (PPP) was prepared within 1 h of blood collection by double centrifugation at 3,500 × g for 15 min at room temperature. Aliquots (0.5 ml) were immediately frozen and stored at −80°C until analysis.

### EV Flow-Cytometry Analysis

PPP was thawed in a water bath for 5 min at 37°C and immediately processed for immunolabeling. PPP was analyzed after a single freeze-thaw cycle. The pre-analytic phase of EV analysis has been previously reported (Campello et al., 2012; Lacroix et al., 2013; Campello et al., 2018). Flow cytometry analysis was performed using a CytoFLEX S flow cytometer (Beckman Coulter, United States), as previously reported (Wisgrill et al., 2016; Campello et al., 2021b). For MV size calibration of the flow cytometer, fluorescent polystyrene beads Gigamix a mix 1:1 of Megamix FSC & SSC Plus (BioCytex, Marseille, France) were used in sizes of 0.1, 0.16, 0.2, 0.24, 0.3, 0.5, and 0.9 μm. Violet side scatter (VSSC) and FL1 channel gain were set to visualize the beads. The side scatter (SSC) from the 405 nm violet laser (VSSC) was used as a trigger signal to discriminate the noise. Gigamix bead solution was gated excluding background noise (because of the solution itself). After turning the set in VSSC and forward scatter (FSC), a rectangular gate was set between the 0.1 and 0.9 μm bead populations and defined as EV gate.

Prior to staining, the antibody mixtures were centrifuged at 20,000 g for 30 min to remove fluorescent particles. The dilution buffer used was sterilized through a 0.2 µm mesh filter to reduce background noise. The final concentration of the antibodies used was from 1 to 5 μg/ml.

Twenty microliters of PPP were stained with 10 μL of calcein-AM 20 μM (referred to calcein-green, Sigma-Aldrich) + 4 μL phycoerythrin (PE)-labelled anti-CD62E antibody (clone HCD62E; BioLegend, San Diego, CA) + 4 µL mouse anti-human-TF (#4509, American Diagnostica) followed by 5 μL anti-mouse IgG secondary antibody Alexa Fluor 647 (1:300 diluted, Thermo Fisher Scientific) + 4 μL PE/Cyanine 7 labelled anti-human CD143 (ACE2, clone 5-369; BioLegend). To confirm that EVs detected by flow cytometry were lipid membrane vesicles the samples were treated with 0.5% Triton X-100 (Sigma-Aldrich) a lipid solubilizing detergent for 10 min at room temperature (RT) and compare with the non-treated samples (Deville et al., 2021).

A second panel consisted of 20 μL of PPP stained with 10 μL of calcein-AM + 4 μL of PE-labelled anti-CD62P antibody (clone CLB-Thromb/6; Beckman Coulter, Marseille, France) + 4 µL ECD labelled anti-CD45 antibody (clone J33; Beckman Coulter) + 4 µL anti-TF followed by mouse Alexa Fluor 647 (see above).

A third panel included 20 μL of PPP stained with 10 μL of calcein-AM + 4 μL of rabbit anti-human- PDGF-β antibody (Thermo Fisher Scientific) + 4 µL anti-TF followed by mouse Alexa Fluor 647 (see above) and anti-rabbit IgG (H + L) PE conjugated (1:300 diluted, Thermo Fisher Scientific) secondary antibodies.

A fourth panel included 20 μL of PPP stained with 10 μL of calcein-AM and 5 μL mouse anti-SARS-CoV-2 nucleoprotein (NP, clone 05, 1:50 diluted, Sino Biological Inc.) followed by 5 μL Alexa Fluor 647 secondary antibody (see above).

One last panel included 20 μL of PPP stained with 5 μL of FITC-labeled Annexin V (Bender MedSystems GmbH, Vienna, Austria) + 5 μL Annexin V-kit binding buffer (Bender MedSystems GmbH).

Calcein-AM staining and the non-conjugated antibodies were incubated for 30 min at 37°C. Subsequently the conjugated antibodies and the secondary antibodies (i.e., anti-mouse IgG Alexa Fluor 647 and anti-rabbit IgG PE), were incubated for 30 min in the dark at RT. Annexin V-staining was incubated for 15 min in the dark at RT.

Parallel incubation was performed with isotype-matched control antibodies and with the secondary antibody alone to exclude non-specific staining. Fluorescence measured with the respective isotype negative control antibody were subtracted in order to avoid non-specific background signal. Stained PPP was then diluted by adding 140 ml of sterile filtered PBS or sterile filtered Annexin V-buffer. EVs were expressed as events/μL with the volume measurement of the CytoFLEX.

Files were exported and data were evaluated by CytExpert (Software Version 1.2, Beckman Coulter).

True EV events were defined as double-positive stained for calcein-AM and one of the following specific antibodies:

- calcein+, total EVs detected
- anti-CD62E, marker of activated endothelial cells (endothelial-derived EVs, E-Selectin+)
- anti‐TF (TF+)
- anti-ACE2 specific endothelial marker (ACE2+)
- anti-CD62P, marker of activated platelets (platelet-derived EVs, P-Selectin+)
- anti-CD45 (leukocyte-derived EVs, CD45^+^)
- anti-PDGF-β specific pericytes marker (PDGF-β+)
- anti-Sars-CoV-2-NP for the detection of virus nucleocapsid protein (Sars-CoV-2-NP+)

Triple-positive EVs were evaluated to ascertain the expression of TF on different EVs subtypes, and namely, calcein-AM, anti-CD62E and anti-TF (E-Selectin+TF+); calcein-AM, anti-ACE2 and anti-TF (ACE+ TF+); calcein-AM, anti-PDGF-β and anti-TF (PDGF-β+TF+); calcein-AM, anti-CD45 and anti-TF (CD45+TF+); calcein-AM, anti-CD62P and anti-TF (P-Selectin+ TF+). Triple-positive calcein-AM, antiCE62E, and anti-ACE2 were also evaluated.

Finally, total phosphatidylserine (PS)+ EVs were evaluated (Annexin V+).

Plasma from 20 healthy subjects (mean age 65 ± 11 years) was also collected and tested over the same study period, as a control group for baseline EV levels. This group consisted of 10 males and 10 females, without history of cardiovascular, autoimmune and acute diseases and not undergoing antithrombotic, antibiotic, or hormonal therapy.

### Phospholipid-Dependent Clotting Time

Procoagulant activity of EVs was measured using STA®-Procoag PPL assay (Diagnostica Stago). The assay measures the clotting time in a system dependent on the procoagulant phospholipid content of the sample (Connor et al., 2009; Campello et al., 2012). The assay is performed using phospholipid-depleted substrate plasma to prevent the influence of any coagulation factors upstream. Factor Xa, in the presence of calcium, triggers the coagulation cascade; a shorter clotting time of the sample indicates increased concentrations of procoagulant phospholipids and thus increased PPL activity which in turn correlates positively with the functional activity of EV present in the sample (Campello et al., 2014).

### EVs Immunofluorescence Staining

To confirm the presence of EVs in plasma, the samples were analyzed also by immuno-fluorescence microscopy. Briefly, 50 µL of PPP were labeled with calcein-AM, mouse anti-human-TF (#4509, American Diagnostica) and rabbit anti-human-ACE2 (#SN0754 at 5 μg/ml final concentration, Thermo Fisher Scientific) for 30 min at 37°C followed by goat anti-mouse IgG Alexa Fluor 647 and donkey anti-rabbit IgG Alexa Fluor 594 secondary antibodies (1:300 diluted, Thermo Fischer Scientific, United States) incubated for 1 h at RT. To analyze EVs bearing virus nucleocapside, PPP was labelled with calcein-AM and mouse Sars-CoV-2-NP incubated for 30 min at 37°C followed by incubation with Alexa Fluor 647 for 1 h at RT. Finally, to analyze the presence of EVs derived from pericytes, PPP was stained with rabbit anti-human PDGF-β and incubation for 30 min at 37°C followed by anti-rabbit IgG Alexa Fluor 594 for 1 h at RT. Subsequently, samples were washed twice with PBS to eliminate unlabeled antibodies and re-suspended in 20 µL of PBS. All the antibodies were diluted in filtered PBS buffer at the same concentration used in cytometry except for ACE2 (see above). Secondary antibodies were also used in the absence of the primary antibodies in order to assess non–specific binding. Finally, labelled EVs (20 µL) were loaded on a clean glass surface covered with 10 μL of Mowiol antifade solution (Sigma-Aldrich). Leica DMI6000CS fluorescence microscope (Leica Microsystem, Wetzlar, Germany) was used and samples were analyzed with differential interference contrast (DIC) and fluorescence objectives. Images were acquired with 100x/1.4 oil immersion lens and using a DFC365FX camera and processed using the Leica Application Suite (LAS-AF) 3.1.1. software (Leica Microsystems).

### Statistical Analysis

Continuous variables were expressed as median and interquartile ranges (IQR) or mean and standard deviation (SD), as appropriate. The normality assumption was assessed by Shapiro-Wilk normality test. Categorical variables were summarized as counts and percentages. Comparisons between groups were performed using nonparametric tests (Mann-Whitney U, Kruskal-Wallis and Friedman test) for quantitative variables and Fisher’s exact tests for frequencies. Associations between continuous variables were analyzed using Spearman’s correlation tests. Logistic regression analyses were performed to determine the associations between clinical outcomes (i.e., VTE, death/worsening and 30-days persistence of symptoms) with EVs levels. ROC curve analysis was performed to evaluate the discrimination threshold of EVs to detect patients at risk for VTE or death/worsening). All tests were 2-sided. Statistical significance was defined as *p* < 0.05. All statistical analyses were performed by GraphPad Prism 7 (GraphPad Software Inc., CA, United States) and MedCalc statistical software for Windows.

## Results

### Characteristics of the Study Population

**FIGURE 1:**
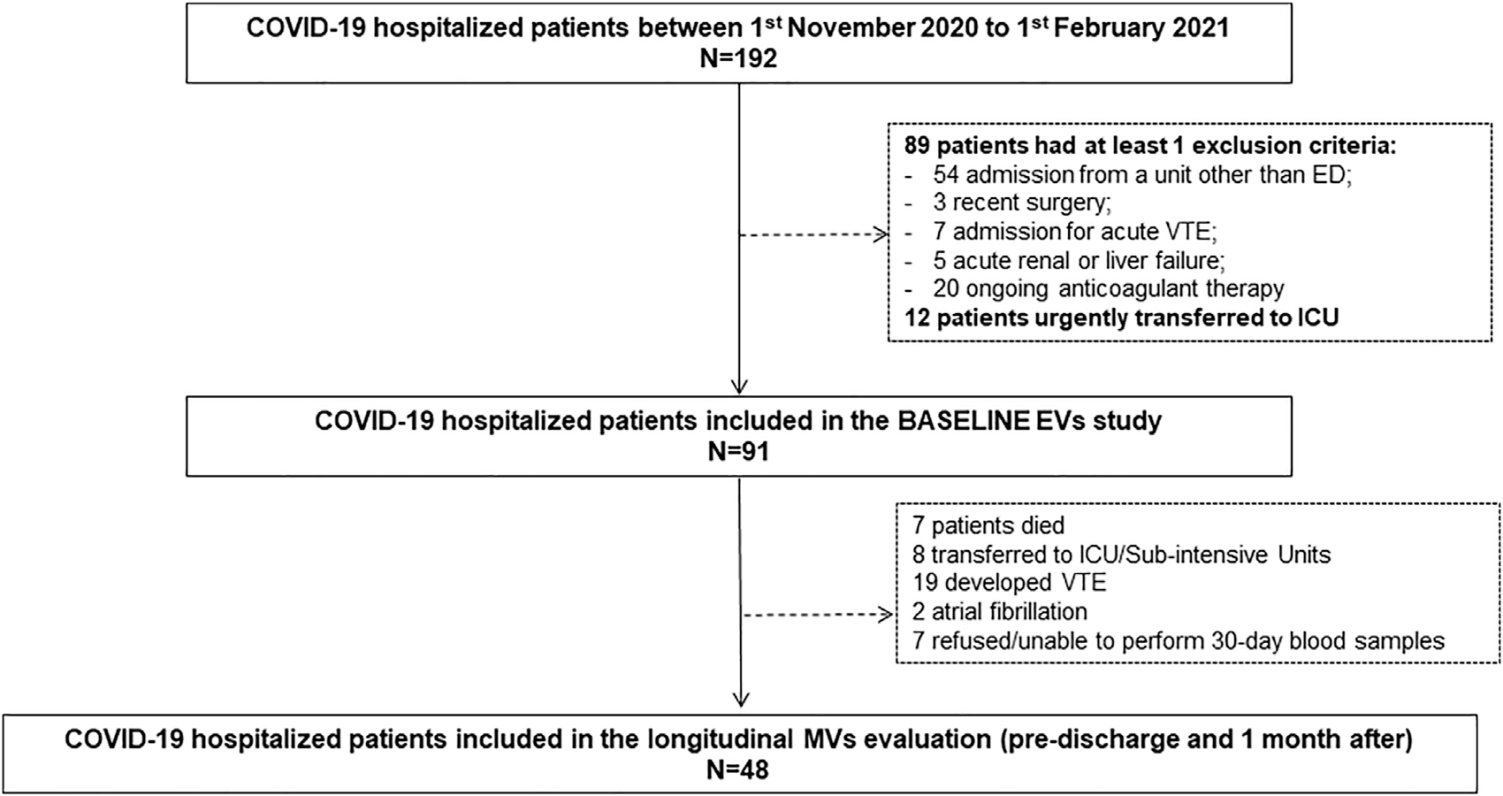
Study flow-chart.

Among 192 patients consecutively admitted to our General Medicine COVID-19 Unit during the study period, 91 were recruited for MV analysis. One hundred and one patients were excluded for the following reasons: 54 for admission from a Department other than ED; 3 for recent surgery; 7 for admission for acute VTE; 5 for acute renal or liver failure; 20 for ongoing anticoagulant therapy; 12 for transfer to the ICU before venous sample collection. Figure 1 shows the study flow-chart.

The mean age of the enrolled patients was 67 ± 14 years, 46 (50.5%) male. Table 1 summarizes the baseline characteristics of the population at time of enrollment and COVID-19 therapeutic protocols, as well as pharmacological thromboprophylaxis.

**TABLE 1:** Baseline characteristics and COVID-19 therapeutic protocols of the study population.

COVID-19 patients n. 91	
Age—years	67 ± 14.0
Gender—F (%)	45 (49.5)
BMI—Kg/m^2^	28.9 ± 6.2
SIC score—n	2.2 ± 1.1
Padua Prediction score—n	3.5 ± 1.7
Comorbidities—n (%)	
Hypertension	54 (59)
Diabetes	20 (22)
Dyslipidemia	24 (26)
Cancer	14 (15)
PaO2/FIO2 (P/F) ratio	255.9 ± 92.7
O_2_ therapy at admission—n (%)	72 (79)
Low-flow (2–5 L/min)	38 (42)
High-flow (≥ 6 L/min)	34 (37)
COVID-19 therapy—n (%)	
Remdesivir	51 (56)
Convalescent Plasma	45 (49)
Steroids	82 (90)
Antibiotics	78 (86)
Pharmacological Thromboprophylaxis	
standard dose	72 (79)
intermediate dose	19 (21)
VTE occurrence—n (%)	19 (21)
Outcome—n (%)	
Discharge	76 (83.5)
Death	7 (7.7)
Worsening	8 (8.8)
Hospital stay—days	10.5 (6–15)
Post-discharge outcome –n (%)^a^	
Persistent symptoms	31 (64.5)
VTE	1 (2)
Re-hospitalization/death	0

Data are shown as mean ± standard or frequency.

BMI, body mass index; SIC, Sepsis-Induced Coagulopathy; VTE, venous thromboembolism events.

a Calculated on 48 patients followed up to 30 days after the discharge.

Among 91 patients enrolled for EV analysis, 7 (7.7%) died, 8 (8.8%) were transferred to the ICU or sub-intensive care units, and 76 (83.5%) were discharged (Table 1). Among 76 discharged patients, 19 developed VTE and 2 atrial fibrillations during hospitalization, 7 declined or were unable to complete the 30-days post-discharge assessment; thus, 48 patients underwent both the pre-discharge and the 30-days post-discharge evaluation (Figure 1). Notably, 31/48 (64.5%) lamented persistent symptoms, mainly asthenia and exertional dyspnea at the 30-days evaluation; one patient (1/48; 2%) developed deep vein thrombosis in the 30 days after the discharge (Table 1).

### Longitudinal Laboratory Evaluation

As shown in Table 2, antithrombin, von Willebrand factor, CRP and ferritin significantly decreased at 30-days post-discharge evaluation. No significant difference was detected in platelet count, D-dimer and factor VIII levels in the longitudinal evaluation.

**TABLE 2:** Laboratory findings in the study population.

COVID-19 admission n. 91		COVID-19 pre-discharge n. 48	COVID-19 30 days n. 48>	P
WBC × 10^3^/L	6.5 [4.4–6.3]	8.8 [7.3–9.7]	5.5 [4.9–7.6]	ns
Platelet count × 10^9^/L	232 [133–324]	305 [248–428]	273 [214–351]	ns
PT ratio	1.00 [0.99–1.01]	1.03 [1.00–1.05]	1.03 [0.98–1.05]	ns
aPTT—sec	24 [20.7–28]	20 [19.7–21]	26 [24.7–26.2]	0.0015
D-dimer—ng/mL	206 [151–729]	213 [150–613]	215 [150–419]	ns
Antithrombin—%	111 [100–117]	118 [114–128]	98 [97–105]	0.012
Fibrinogen—g/L	4.7 [3.9–5.2]	4.6 [3.6–4.9]	3.9 [3.5–4.7]	ns
Factor VIII—%	189 [137–277]	286 [179–367]	139 [119–216]	ns
VWF Ag—%	304 [239–384]	—	223 [157–248]	0.007
CRP—mg/L	56 [28–118]	9.9 [6.2–12]	2.9 [2.9–7]	<0.001
Ferritin—μg/L	663 [473–1476]	555 [395–985]	165 [144–379]	<0.001
Interleukin 6—ng/L	23 [18–71]	17 [14–32]	14.8 [10–18]	ns

Data are shown as median and range interquartile. P are calculated using Friedman test.

Reference ranges: WBC 4.40–11.00; platelet count 150–450; PT ratio 0.9–1.20; aPTT 22–32; D-dimer 0–350; antithrombin 80–120; fibrinogen 1.50–4.50; factor VIII 60–160; VWF antigen 60–160; CRP 0–6.0; interleukin (IL)-6 0–5.9; ferritin 20–250.

aPTT, activated partial thromboplastin time; CRP, C-reactive protein; PT, prothrombin time; WBC, white blood cells; VWF, von Willebrand factor.

### Baseline and Longitudinal EVs Evaluation

Plasma concentrations of PS + EVs (identified by Annexin v+), endothelium-derived (identified by E-Selectin+ and ACE2+), platelet-derived (identified by P-Selectin+), leukocyte-derived (identified by CD45^+^) EVs, TF+, leukocyte-derived expressing TF (CD45+TF+), PDGF-β+ as well as SARS-CoV-2-NP+ were significantly increased in COVID-19 patients at baseline vs. healthy controls. PPL (functional test to measure the procoagulant activity of EVs, sec) was significantly shorter in COVID patients vs. healthy subjects (Table 3).

**TABLE 3:** EVs levels COVID-19 patients and healthy controls.

EVs subgroup	COVID-19 baseline (n. 91)	Healthy controls (n. 20)	*p*
Calcein+	2409 [1527–3248]	1145 [809–1884]	0.02
Annexin V+	1856 [1482–2490]	1009 [871–1207]	<0.0001
E-Selectin+	620 [471–876]	317 [232–388]	<0.0001
P-Selectin+	1054 [502–1682]	848 [476–972]	0.03
CD45+	458 [25–807]	230 [192–315]	<0.0001
TF+	60 [26–90]	38 [14–57]	0.02
CD45+TF+	52 [33–88]	15 [10–34]	0.02
ACE2+	969 [540–1314]	334 [284–923]	<0.0001
E-Selectin+TF+	69 [46–96]	52 [49–65]	0.04
E-Selectin+ACE2+	518 [394–727]	286 [223–384]	<0.0001
E-Selectin+ACE2+TF+	59 [34–88]	—	—
PDGF-β+	102 [48–197]	79 [54–100]	0.04
PDGF-β+TF+	40 [21–73]	26 [17–35]	0.007
SARS-CoV-2-NP+	218 [78–390]	10.5 [7–27]	<0.0001
PPL—sec	46.7 [36.4–51.9]	52 [45–62]	0.006

Data are shown as median and range interquartile.

EVs, extracellular vesicles; TF, tissue factor; PDGF-β, Platelet-derived growth factor-subunit B; ACE, Angiotensin-converting enzyme-2; PPL, phospholipid-dependent clotting time.

**FIGURE 2:**
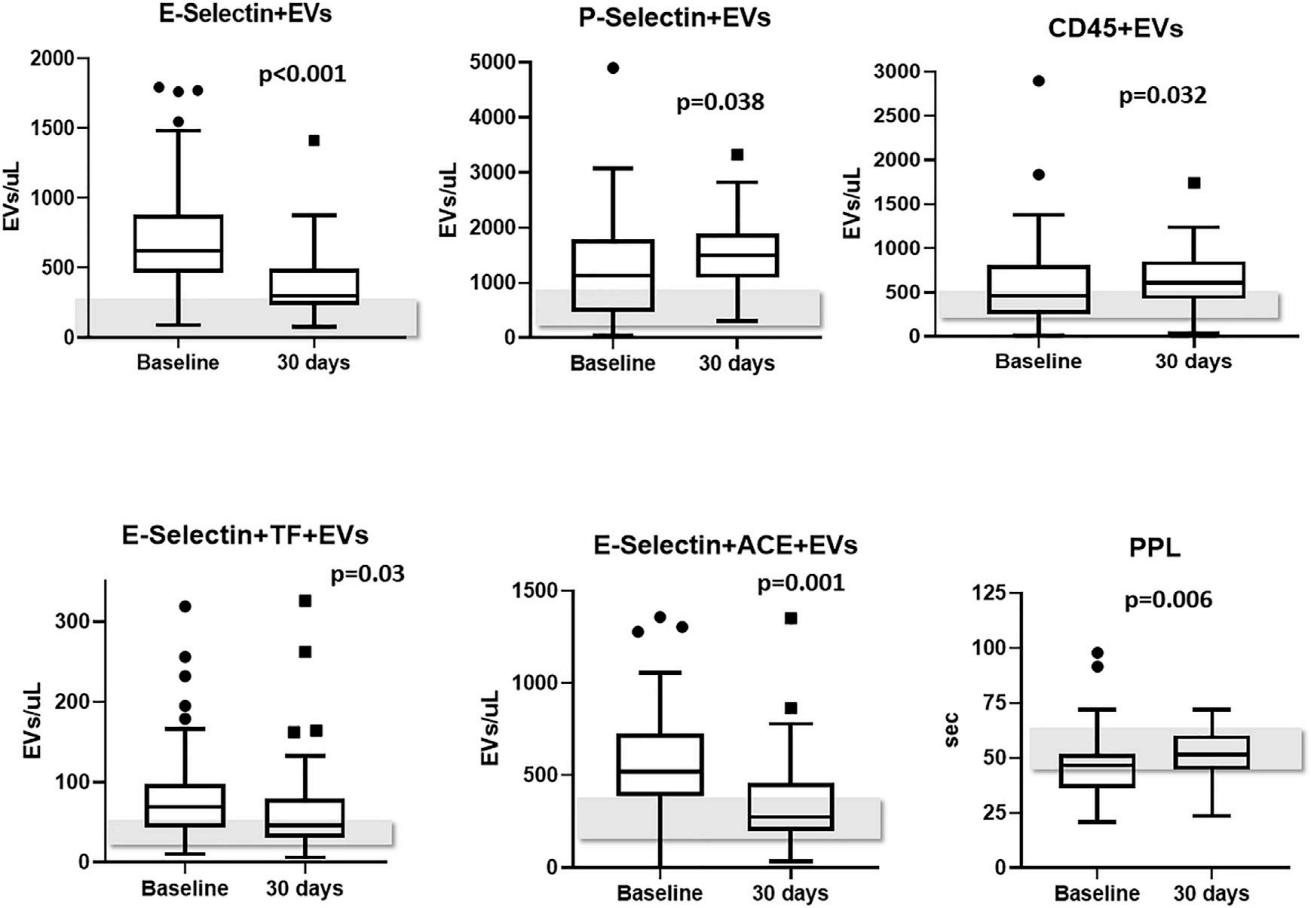
EVs subtypes significantly changed from baseline to 30-days follow-up. Levels of EVs measured in healthy controls are reported as grey bars; Abbreviations: EVs: extracellular vesicles; ACE2: angiotensin-converting enzyme-2.

Levels of PS + EVs and endothelium-derived EVs showed a significant decrease from baseline to 30-days post-discharge evaluation (−654 and −286 EVs/μL, respectively, *p* < 0.001). Likewise, plasma concentrations of leukocyte-derived expressing TF (CD45+TF+), endothelium-derived EVs expressing TF (E-Selectin+TF+) and endothelium-derived EVs expressing ACE2 (E-Selectin+ACE2+) also decreased from baseline to 30-days post-discharge evaluation (*p* = 0.001, *p* = 0.03, and *p* = 0.001, respectively). On the other hand, levels of platelet-derived EVs (P-Selectin+) and of leukocyte-derived EVs (CD45^+^) significantly increased from baseline to 30-days post-discharge (*p* = 0.038 and *p* = 0.032, respectively). Levels of TF+, ACE2+, PDGF-β+, and SARS-CoV-2-NP + EVs did not change significantly during the monitoring (Table 4). It bears noting that PPL increased significantly from baseline to 30-days post-discharge evaluation (+ 6.3 s, *p* = 0.006), meaning an overall reduced presence of procoagulant EVs in the plasma (Table 4). Figure 2 shows levels of EVs significantly changed during follow-up.

**TABLE 4:** EVs levels in the study population.

EVs subgroup	Baseline	Pre-discharge	30 days post-discharge	Change over study period^a^	*p* (baseline vs. 30 days)
Calcein+	2409 [1527–3248]	2286 [1561–3942]	1675 [1041–2495]	−633 [-1053, −288]	0.005
Annexin V+	1856 [1482–2490]	1822 [1193–2417]	1295 [850–1457]	−654 [−1069, −254]	0.0005
E-Selectin+	620 [471–876]	641 [386–1124]	298 [237–489]	−286 [−379, −176]	<0.001
P-Selectin+	1054 [502–1682]	1126 [480–1777]	1495 [1091–1874]	371 [25, 742]	0.038
CD45+	458 [25–807]	498 [259–773]	605 [432–849]	124 [11,243]	0.032
TF+	60 [26–90]	61 [24–112]	65 [41–98]	15 [−1.30]	Ns
CD45+TF+	52 [33–88]	51 [35–96]	31 [20–81]	−21 [−20,−9]	0.001
ACE2+	969 [540–1314]	1022 [461–1489]	808 [562–1011]	−109 [−296.89]	Ns
E-Selectin+TF+	69 [46–96]	63 [27–128]	46 [31–79]	−17 [−2, −31]	0.03
E-Selectin+ACE2+	518 [394–727]	505 [337–825]	274 [203–450]	−229 [−320, −130]	0.001
E-Selectin+ACE2+TF+	59 [34–88]	55 [324–100]	44 [28–76]	−11 [−26.3]	Ns
PDGF-β+	102 [48–197]	72 [48–121]	89 [63–128]	−12 [−45.15]	Ns
PDGF-β+TF+	40 [21–73]	42 [24–72]	41 [25–63]	1 [−10.11]	Ns
SARS-CoV-2-NP+	218 [78–390]	226 [79–324]	174 [128–322]	−24 [−99.82]	Ns
PPL—sec	46.7 [36.4–51.9]	43.8 [37.9–52.1]	51.5 [45–60.2]	6.3 [2–11]	0.006

Data are shown as median and range interquartile.

a Change over period is calculated between baseline and 30-days post-discharge evaluation.

EVs, extracellular vesicles; TF, tissue factor; PDGF-β, platelet-derived growth factor-subunit B; ACE, Angiotensin-converting enzyme-2; PPL, phospholipid-dependent clotting time.

As regards the procoagulant potential of EVs, we showed that PS + EVs strongly correlated with PPL shortening (r −0.74, *p* < 0.0001); Calcein+, E-Selectin+, P-Selectin+, ACE2+, E-Selectin+ACE2+, and CD45^+^ correlated weakly but significantly with a shorter PPL. On the other hand, TF+, CD45+TF+, E-Selectin+ACE2+TF+, PDGF-β+, and SARS-CoV-2-NP+ did not correlate with PPL (Table 5).

**TABLE 5:** Correlations between EV levels and functional EV test (PPL).

EVs subgroup	R	P
Calcein+	−0.31	0.016
Annexin V+	−0.74	<0.0001
E-Selectin+	−0.52	0.003
P-Selectin+	−0.51	0.005
CD45+	−0.44	0.04
TF+	−0.15	Ns
ACE2+	−0.47	0.019
CD45+TF+	−0.19	Ns
E-Selectin+TF+	−0.12	Ns
E-Selectin+ACE2+	−0.47	0.021
E-Selectin+ACE2+TF+	−0.19	Ns
PDGF-β+	−0.26	Ns
PDGF-β+TF+	−0.29	Ns
SARS-CoV-2-NP+	−0.22	Ns

Data are shown as median and range interquartile.

EVs, extracellular vesicles; TF, tissue factor; PDGF-β, Platelet-derived growth factor-subunit B; ACE2, Angiotensin-converting enzyme-2; PPL, phospholipid-dependent clotting time.

Regarding correlations between EVs levels and inflammation parameters, we showed that only leukocyte-derived EVs positively correlated with IL-6 (r 0.41, *p* = 0.013), CRP (r 0.37, *p* = 0.02), FVIII (r 0.38, *p* = 0.03), VWF (r 0.55, *p* = 0.0005) and white blood cells (r 0.48, *p* = 0.0011). Leukocyte-derived EVs expressing TF positively correlated as well with IL-6 (r 0.57, *p* = 0.024) and CRP (0.37, *p* = 0.04). These correlations were confirmed in all the three times of monitoring (admission, pre-discharge and 30 days).

### Correlations Between EV Levels and Venous Thromboembolism or Prognosis

**FIGURE 3:**
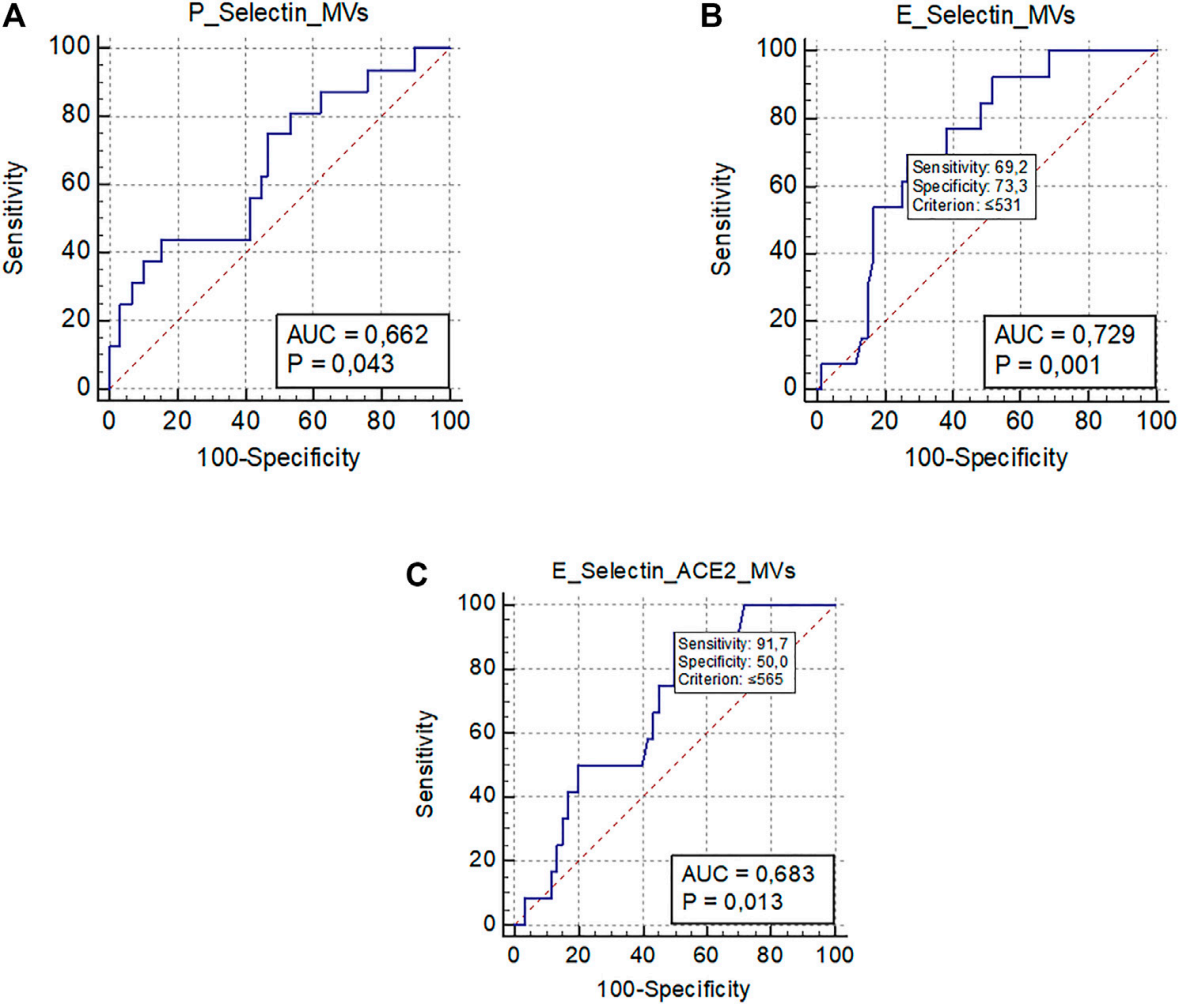
ROC curve analysis representing significant associations between MVs levels and clinical outcomes. **(A)** Association between platelet-derived MVs (P-Selectin + MVs) and venous thromboembolism; **(B)** Association between endothelial-derived MVs (E-Selectin + MVs) and worsening/death; **(C)** Association between endothelial-derived MVs expressing ACE2 (E-Selectin+ACE2+ MVs) and worsening/death.; Abbreviations: ROC: receiver operating characteristic; MVs: microvesicles; ACE2: angiotensin-converting enzyme-2; AUC: area under the curve.

As shown in Table 1, VTE occurred in 19 patients (21%) during the hospitalization, despite pharmacological thromboprophylaxis. Supplementary Table S1 included types and site of occurred VTE. Baseline levels of platelet-derived EVs were significantly associated with the development of VTE [OR 1.07 (95% CI 1.00–1.013); *p* = 0.024]. A ROC curve analysis showed that levels of P-Selectin + EVs >1,054/µL were significantly associated with the development of VTE (AUC 0.66, *p* = 0.043) (Figure 3A). However, sensitivity and specificity were quite low due to the small sample of VTE patients.

Finally, 76 (83.5%) patients were discharged, and 15 (16.5%) died or worsened during the hospitalization (Table 1). The only EV subtype significantly associated with a better prognosis were E-Selectin + EVs [OR 0.997 (95% CI 0.995–0.999); *p* 0.026] and E-Selectin+ACE2+EVs [OR 0.997 (95% CI 0.994–0.999); *p* 0.042]. The ROC curve analysis confirmed that plasma concentrations of E-Selectin + EVs ≤531/µL were significantly associated with a bad outcome (worsening or death) (AUC 0.72, *p* < 0.001), as were plasma concentrations of E-Selectin+ACE2+EVs ≤565/µL (AUC 0.68, *p* = 0.013) (Figures 3B,C).

We detected no association between pre-discharge EV levels and any of the COVID-19 therapies administered (convalescent plasma or remdesivir).

We detected a significant association between 30-days concentration of P-Selectin + EVs [OR 1.003 (95% CI 1.0017–1.0043); *p* < 0.0001] and of CD45 + EVs [OR 1.005 (95% CI 1.0027–1.0074); *p* < 0.0001] and persistence of symptoms.

### Immunofluorescence Microscopy

**FIGURE 4:**
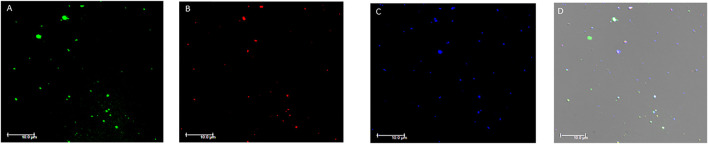
Fluorescence microscopy images of plasma extracellular vesicles. **(A)** Calcein-AM FITC staining (green fluorescence); **(B)** anti-human TF staining (red fluorescence); **(C)** anti-human ACE2 staining (blue fluorescence). **(D)** Overlay of the three fluorescence images and differential interference contrast (DIC). MVs were isolated from plasma and images acquired by fluorescence microscopy (Leica DMI6000CS, 100x/1.4 oil immersion objective) using a DFC365FX camera and LAS-AF 3.1.1. software. Scale bar 10 μm; Abbreviations: FITC: Fluorescein; TF: tissue factor; ACE2: angiotensin-converting enzyme-2.

The analysis with immunofluorescence microscopy allowed us to confirm the presence of EVs in the plasma samples tested by flow cytometry. The immunostaining demonstrated the presence of EVs labelled with the different antibodies used in flow cytometry. EVs stained positive for one or more markers at the same time confirming what we observed in the flow cytometry analysis. Particularly, the expression of TF+, ACE2+, SARS-CoV-2-NP+ and PDGF-β+ EVs was confirmed (Figure 4 and Supplementary Figures S1, S2).

## Discussion

The peculiarity of this study is that we characterized circulating EVs derived from cells involved in COVID-19-associated coagulopathy (Connors and Levy, 2020; Lippi et al., 2021). Additionally, for the first time, EV levels were longitudinally measured at admission, discharge and 30 days after discharge. We observed high plasma concentrations of endothelium-derived EVs at admission which remained elevated at discharge, but decreased significantly 1 month later. Particularly, concentrations of endothelium-derived EVs were determined by E-Selectin staining, and further characterized by E-Selectin and ACE2 double staining, and E-Selectin and TF double staining. All these EV subtypes showed the same longitudinal trend.

Endothelial injury and/or dysfunction appears to be the main driver in COVID-19-associated coagulopathy (Dupont et al., 2021). SARS-CoV-2 enters endothelial cells *via* angiotensin converting enzyme 2 (ACE2) expressed on the surface of arterial and venous endothelial cells and arterial smooth muscle cells (Hamming et al., 2004). The cytopathic effect from the direct viral infection of these cells likely induces endothelial activation and the impairment of the intrinsic antithrombotic properties of endothelial cells (Varga et al., 2020). We confirmed the presence of circulating EVs derived from endothelial cells expressing ACE2, as well as EVs derived from activated endothelial cells expressing TF. Though the endothelial injury caused by the SARS-CoV-2 infection persisted at discharge, it was significantly reduced 30 days later.

In COVID-19-associated coagulopathy, the endothelial injury is rapidly followed by a series of events culminating with platelet activation, adhesion to the injured subendothelial matrix and pro-aggregation (Iba et al., 2020a; Lippi et al., 2021; Iba et al., 2020b; Connors and Levy, 2020; Zhang et al., 2020). We confirmed high concentrations of platelet-derived EVs in COVID-19 patients, which remained elevated at discharge and further increased 1 month later. This finding was quite peculiar and indicates that the process of platelet activation induced by the virus may persist long after complete recovery and negativization (Dixit et al., 2021). The plasma concentrations of leukocyte-derived EVs followed the exact same trend. Neutrophil activation is another essential mechanism underlying the common finding of a prothrombotic state in COVID-19 patients (Iba et al., 2020a; Lippi et al., 2021; Iba et al., 2020b; Connors and Levy, 2020). Neutrophils can be colonized by SARS-CoV-2 by internalization through the Fc receptor or activated by endothelial cells, platelets, and monocytes/macrophages, thus triggering the release of neutrophil extracellular traps (NETs), which can directly activate the intrinsic pathway of blood coagulation (Gould et al., 2014; Ackermann et al., 2021). In addition, activated platelets in COVID-19 patients show a high propensity to form platelet-leukocyte aggregates (Hottz et al., 2020; Zaid et al., 2020). In fact, both P-Selectin expression and the number of platelet-neutrophil and platelet-monocyte aggregates were found to be significantly enhanced in COVID-19 patients (Manne et al., 2020). We confirmed that activated platelets and leukocytes can shed microvesicles as a result of being activated and that cellular activation lasts long after complete recovery and negativization (Dixit et al., 2021). Additionally, only levels of leukocyte-derived EVs were shown to correlate with inflammation markers, confirming a role for this EV subtype in acute and chronic inflammation (Campello et al., 2016b).

The phospholipid-dependent clotting time (PPL, sec) confirmed the presence of an increased procoagulant activity—as reflected by a shorter PPL—due to circulating phospholipids in COVID-19 patients at admission, with a slight further shortening at discharge, versus a significant prolongation 30 days after discharge. This means that the overall procoagulant activity associated with the presence of circulating EVs was still present at discharge, but was resolved 30 days after. We observed that PPL greatly correlated with PS + EVs, indicating that these EVs convey the strongest phospholipid-dependent procoagulant activity. Additionally, PPL more weakly but significantly correlated also with endothelium-derived (both E-Selectin and ACE+)EVs, platelet-derived and leukocyte-derived EVs, indicating that these EV subtypes may convey phospholipid-dependent procoagulant activity as well. It is critical to note that other EV subtypes did not correlate with PPL, thus excluding any non-phospholipid-dependent procoagulant or clotting activity (e.g., TF-driven).

One additional mechanism of COVID-19 coagulopathy is the continuous release of TF in the pulmonary circulation, and in the blood vessels of other organs and tissues, thus contributing to the activation of secondary hemostasis (Grover and Mackman, 2018; Iba et al., 2020a; Lippi et al., 2021; Iba et al., 2020b; Connors and Levy, 2020). The release of TF stems mainly from extensive endothelial damage, as mentioned earlier. In fact, we found increased concentrations of endothelium-derived EVs expressing TF+ at admission and discharge, with a significant decrease at 30-days post-discharge. Additionally, we described the same figure with leukocyte-derived EVs expressing TF+, being increased at admission and discharge, with a significant decrease at 30-days post-discharge. Interestingly, while levels of leukocyte-derived EVs (CD45^+^) increased 30-days post discharge, leukocyte-derived EVs expressing TF significantly decrease. This observation may indicate that the amount of activated leukocytes decreases 1 month after the viral infection and the pathological inflammatory response is dwindling, probably due to the recovery of endothelial dysfunctional disease, persisting a sort of global inflammatory activation though. Our findings indicate that EVs originated from injured endothelial cells and activated leukocytes carry TF on their surface.

On the other hand, we observed no difference in TF + EVs, stained with CD142 but negative for endothelial or leukocyte markers, during the monitoring period. We did not investigate other possible origins of TF + EVs (i.e., platelets) which may account for this finding. It is worth mentioning that there is some controversy surrounding the use of flow cytometry for TF + EVs detection, since the suggested method to measure TF + MVs is EV-associated TF activity (Nieuwland et al., 2019; Mackman et al., 2021). Rosell A. et al. reported that EV-TF activity was significantly higher in patients with COVID-19 vs. healthy controls (Rosell et al., 2021). Moreover, Guervilly et al. showed increased MV-TF activity in patients with severe COVID-19 vs. moderate disease (Guervilly et al., 2021). Our results confirm that flow cytometry may not be the best method to measure “total” TF + EVs, though it may be used to determine the cell origin of EVs expressing TF.

We also measured plasma concentrations of EVs derived from activated pericytes using PDGF-β antibody. Pericytes are multi-functional mural cells embedded at intervals within the walls of capillaries throughout the body (Winkler et al., 2010; Armulik et al., 2005). Some evidence suggests that pericytes and microvascular smooth muscle cells express higher concentrations of SARS-CoV-2 receptor ACE2 than endothelial cells, and may play a key role in the pathogenesis of COVID-19 coagulopathy following SARS-CoV-2-mediated endothelial injury (He et al., 2020). Intact pericytes appear to limit endothelial pro-thrombotic responses. We found increased plasma concentrations of PDGF-β+MVs in COVID-19 patients that slightly decreased 30 days after discharge, which may suggest the activation and subsequent vesiculation of pericytes.

Finally, we measured SARS-CoV-2-NP + EVs, defined as EVs expressing SARS-CoV-2 nucleocapsid protein, its most abundant protein (Das and Suresh, 2006). Research suggests that its functions include the enhancement of virion assembly and viral transcription by forming complexes with genomic RNA (McBride et al., 2014). Antibodies against the nucleocapsid protein are the most sensitive marker for serologic diagnosis of SARS-CoV-2 infection (Burbelo et al., 2020). COVID-19 patients showed high concentrations of SARS-CoV-2-NP + EVs that remained elevated up to 30 days after discharge. Notably, the plasma concentrations of these EV subtypes did not correlate with PPL. These findings indicate that EVs bearing the nucleocapsid protein do exist and may play a role in the viral cellular spreading; however, they do not appear to exert any phospholipid-dependent procoagulant activity.

A recent study by Balbi et al. described the antigenic profile of circulating EVs in COVID-19-patients (Balbi et al., 2021). The Authors reported that a combination of seven surface molecules (CD49e, CD209, CD86, CD133/1, CD69, CD142, and CD20) was able to cluster COVID (+) versus COVID (−) patients and healthy subjects; in addition, TF (CD142) was one of the most expressed EV surface marker in positive vs. negative SARS-CoV-2 patients. However, concentrations of EVs were analyzed using a commercial kit that detected exosomes and results are difficult to compare with EV detection by flow cytometry. The Authors confirmed high EV-TF activity in COVID-19 patients (Balbi et al., 2021).

As for the possible clinical significance of EVs, baseline levels of platelet-derived EVs were significantly associated with the occurrence of VTE complications. Although OR was low due to the small number of events (n. 19), a ROC analysis revealed a possible cut-off of P-Selectin EVs associated with a higher thrombotic risk. Previous reports showed that EV-TF activity was significantly increased in COVID-19 patients with symptomatic clinical thromboembolic events13. Our study also demonstrated the important role of activated platelets in the pathogenesis of immunothrombosis caused by SARS-CoV-2. The relative high incidence of VTE complications in our cohort may be due to the systematic use of Doppler ultrasound of lower limbs during hospitalization, as a similar incidence has been reported in studies undergoing systematic ultrasonography in COVID-19 patients Avruscio et al., 2020; Nopp et al., 2020).

In addition, we uncovered a protective role of high concentrations of endothelium-derived EVs (both E-Selectin+ and E-Selectin+ACE2+) which resulted significantly associated with death or worsening, if decreased. Although the number of events was low (n. 15) to draw any definitive conclusions, we hypothesized that this EV subtype may act as pro-inflammatory mediators and thus helping contain viral spreading. Kudryavtsev I. also showed that reduced levels of endothelial-EVs (i.e., CD146+) were detected in patients with severe infection (Kudryavtsev et al., 2021). Finally, we detected an association between 30-days levels of platelet- and leukocyte-derived EVs and the presence of symptoms, indicating the persistence of cellular activation due to thrombo-inflammation as the possible underlying cause of the persistent illness.

One of the strengths of our study lies in the measurement of EV subtypes derived from cells involved in COVID-19 coagulopathy using high sensitivity flow cytometry, as well as the longitudinal evaluation of EVs. We also performed a functional test to ascertain the procoagulant potential of EVs. Finally, we confirmed the presence of plasma EVs using fluorescence microscopy stained with the same primary and secondary antibodies used in flow cytometry.

We would be remiss if we did not acknowledge some of the limitations of our study. Firstly, despite a substantial number of baseline samples, the number of longitudinal measurements was low. Moreover, the low number (n. < 20) of clinical events (VTE and death/worsening) means that associations should be tempered. Additionally, we only enrolled COVID-19 patients with mild-to-moderate disease. As EVs are an extremely sensible parameter to cells activation or apoptosis and are influenced by the presence of severe coagulopathy as well as by different therapeutic protocols, we decided to focus on a population as homogeneous as possible in terms of clinical characteristics, therapeutic protocols, thromboprophylaxis regimen and potential complications. Thus, these results are not generalizable to the whole SARS-CoV-2 spectrum of infections. Secondly, we did not thoroughly evaluate the possible origins of TF + EVs (e.g., EVs expressing TF and P-Selectin); however, we accounted for endothelium-derived EVs expressing TF, ACE2+ expressing TF, CD45^+^ and PDGF-β+ expressing TF, in an attempt to further explain the endothelial injury caused by SARS-CoV-2 infection (Canzano et al., 2021). Thirdly, as previous reports confirmed increased EV-TF activity in COVID-19 patients, we chose to perform a different functional test to evaluate the procoagulant activity associated with EV phospholipids.

In conclusion, our study confirmed the presence of increased levels of several EV subtypes derived from cells involved in COVID-19 coagulopathy at admission and discharge in COVID-19 patients admitted to a medical Unit. We also described the presence of EVs derived from activated pericytes, as well as EVs expressing SARS-CoV-2-nucleocapsid protein. A trend analysis showed that levels of endothelium-derived EVs, endothelium-derived EVs expressing ACE2 and TF, and leukocyte-derived EVs expressing TF decreased 30 days after discharge. However, platelet-EVs and leukocyte-EVs continue to increase 30 days after discharge, indicating that cellular activation persists long after the acute phase. We also showed that only PS+, endothelium-, platelet- and leukocyte-EVs exerted phospholipid-dependent clotting activity. Finally, higher plasma concentrations of platelet-derived EVs may be associated with VTE complications, whereas reduced plasma concentrations of endothelium-derived EVs may be associated with adverse outcomes. Larger studies are needed to refine our understanding of the role of the different EV subtypes in the pathogenesis of COVID-19 coagulopathy and the related clinical complications.

## Data Availability

The raw data supporting the conclusion of this article will be made available by the authors, without undue reservation.

## Abbreviations

ED, emergency department; EVs, extracellular vesicles; ICU, intensive care unit; VTE, venous thromboembolism.
