# Supplementary material for: Longitudinal Trend of Plasma Concentrations of Extracellular Vesicles in Patients Hospitalized for COVID-19
Source: Front Cell Dev Biol. 2022 Jan 17;9:770463. doi: 10.3389/fcell.2021.770463 (PMC8801799; doi:10.3389/fcell.2021.770463)
Supplement: Supplementary file 1 [file Table1.DOCX]

Supplementary Material

**Longitudinal trend of plasma concentrations of extracellular vesicles in patients hospitalized for COVID-19**

**Supplementary table 1. Characteristics of VTE events occurred in the study population.**

|  | **VTE events**  n. 19 |
| --- | --- |
| **Age –** years | 65 ± 14 |
| **Sex –** M(%) | 9 (47%) |
| **Pulmonary embolism –** n(%) | 3 (15.8%) |
| **Pulmonary embolism and DVT** – n(%) | 1 (5.3%) |
| **Symptomatic proximal DVT** – n (%) | 2 (10.5%) |
| **Asymptomatic proximal DVT –** n(%) | 2 (10.5%) |
| **Symptomatic distal DVT –** n (%) | 3 (15.8%) |
| **Asymptomatic distal DVT –** n (%) | 3 (15.8%) |
| **Catheter-related DVT –** n (%) | 5 (26.3%) |

VTE: venous thromboembolism; DVT: deep vein thrombosis; M: male

Symptomatic: DVT diagnosed after patients symptoms or sudden D-dimer increase.

Asymptomatic: DVT diagnosed by lower limb ultrasonography without any symptom or D-dimer increase.

**Supplementary Figure 1.** **Fluorescence microscopy images of plasma extracellular vesicles**


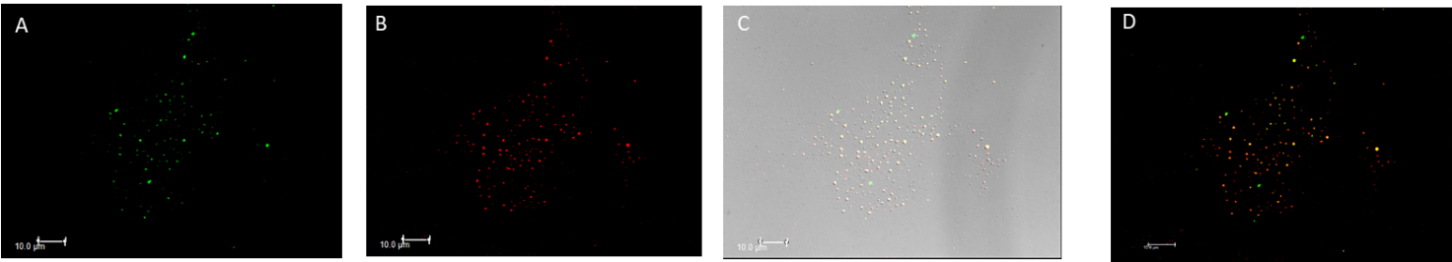


A: Calcein-AM FITC stainig (green fluorescence); B: anti-human SARS-CoV-2-NP staining (red fluorescence); C: merged image of Calcein-AM, SARS-CoV-2-NP fluorescence and differential interference contrast (DIC); D: overlay of fluorescence images.

Scale bar 10 µm

Abbreviations: FITC: Fluorescein; NP:nucleoprotein.

**Supplementary Figure 2. Fluorescence microscopy images of plasma extracellular vesicles**


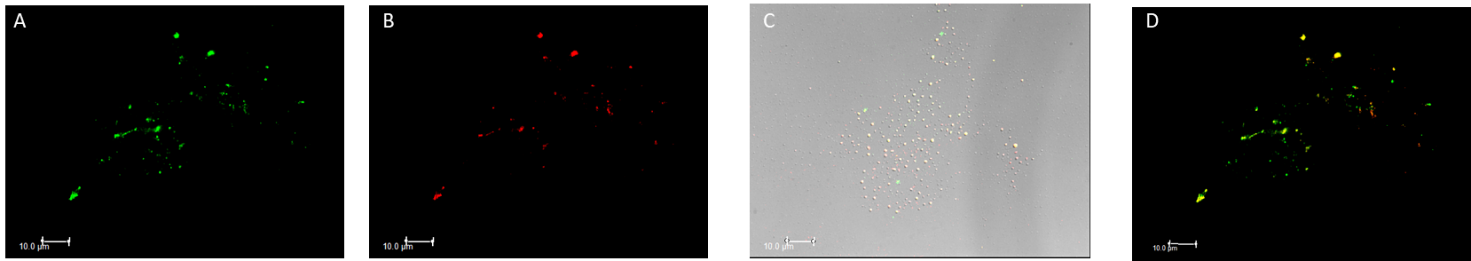


A: Calcein-AM FITC staining (green fluorescence); B: anti-human PDGF-β staining (red fluorescence); C: overlay image of fluorescences and differential interference contrast (DIC); D: merged fluorescence images. Scale bar 10 µm.

Abbreviations: FITC: Fluorescein; platelet-derived growth factor receptor-β (PDGF-β).
